# Be quiet and man up: a qualitative questionnaire study into fathers who witnessed their Partner’s birth trauma

**DOI:** 10.1186/s12884-020-02902-2

**Published:** 2020-04-22

**Authors:** Emily Daniels, Emily Arden-Close, Andrew Mayers

**Affiliations:** Bournemouth University, Bournemouth, England

**Keywords:** Birth trauma, Fathers’ mental health, Perinatal, Paternal mental health, PTSD, Qualitative

## Abstract

**Background:**

Research focusing on paternal mental health is limited, especially regarding the impact of the experience of poor mental health in the perinatal period. For example, little is known about the experiences of fathers who witness their partner’s traumatic birth and the subsequent impact on their mental health. Therefore, the aim of this study was to explore fathers’ experiences of witnessing a traumatic birth, how these experiences impacted their wellbeing, and what support they received during and following the traumatic birth.

**Methods:**

Sixty-one fathers were recruited via targeted social media to complete an anonymous online qualitative questionnaire regarding their birth trauma experience. Eligible participants were fathers aged eighteen or over, resided in the UK and had witnessed their partner’s traumatic birth (that did not result in loss of life). Thematic analysis was used to analyse the questionnaire data.

**Results:**

Three main themes were identified: ‘fathers’ understanding of the experience’ (subthemes: nothing can prepare you for it; merely a passenger; mixed experiences with staff; not about me); ‘life after birth trauma’ (subthemes: manhood after birth; inability to be happy; impact on relationships); and ‘the support fathers received vs what they wanted’ (subthemes: prenatal support; birth support; and postnatal support).

**Conclusions:**

Fathers reported that witnessing their partner’s traumatic birth had a significant impact on them. They felt this affected their mental health and relationships long into the postnatal period. However, there is no nationally recognised support in place for fathers to use as a result of their experiences. The participants attributed this to being perceived as less important than women in the postnatal period, and maternity services’ perceptions of the father more generally. Implications include ensuring support is available for both the mother and father following a traumatic birth, with additional staff training geared towards the father’s role.

## Background

Concern for maternal mental health has risen in recent years, as shown by the expansion in research and public interest around the causes and impact of poor maternal mental health on the mother and her family. The mother’s mental health during pregnancy and up to a year postnatally is crucial not just to her wellbeing, but also the mental health of her partner [1]. Additionally, post-traumatic stress disorder (PTSD) following birth trauma in women is a newly emerging area of research, with evidence suggesting that 3.17% of women report post-traumatic stress symptoms following childbirth [2]. While research exists about the impact of birth trauma on mothers (e.g. Modarres, Afrasiabli, Rahnama & Montazeri, [3]), little is known about the impact on fathers. The current study addresses this gap by exploring fathers’ perceptions of witnessing their partner’s traumatic birth. For this paper, ‘birth trauma’ is defined as physical and emotional suffering during birth that resulted from either complications, physical injury or negative reactions during the birthing experience [4]. Examples include, but are not limited to, sudden changes to the birth plan, emergency caesarean, post-birth complications and inadequate care received from staff. A feeling of lack of control around the unfolding of the birth may also increase the likelihood of its being perceived as traumatic [5].

In a meta-ethnographic study into mothers’ experiences of birth trauma, Elmir, Schmied, Wilkes and Jackson [6] identified that these women feel invisible and out of control. They highlighted the need for women to be fully informed and included in decision-making in order to increase their sense of control, which could in turn reduce perceived trauma. In light of this, we need to examine whether fathers (who witness their partner’s traumatic birth) have similar experiences. Shaban et al.’s [4] definition of birth trauma is often used to describe women’s experiences. However, a study of post-traumatic stress and depression in 212 couples following the birth of their child found that fathers appeared to mirror their partners responses, especially regarding symptoms of post-traumatic stress [7]. Posttraumatic stress symptoms can have significant consequences for parents’ wellbeing and ability to function postnatally [7]. Crucially, in recent years, fathers are more likely to be present at their child’s birth than has traditionally been the case. If that birth becomes traumatic, fathers are potentially witnessing (often helplessly) the potential risk of physical harm and/or death of their partner and/or child. Current evidence tells us very little about fathers’ experiences of witnessing that trauma, the impact that this may have on them, and the support they receive to help them deal with such trauma.

Limited research has addressed the impact of traumatic birth on fathers. Etheridge and Slade [8] in a qualitative study, examined the impact of their partners’ birth trauma in 11 fathers. They found that fathers experienced birth as a rollercoaster of emotions, characterised by ‘isolation and abandonment’. Their findings that fathers can experience trauma as a result of childbirth may arise in part due to the current maternity model, where the mother tends to be the sole focus as the patient. Participants reported feeling unjustified in their feelings and coped by avoiding discussion of the events of the birth trauma and their emotions surrounding that. In a qualitative study with 20 fathers who had witnessed the resuscitation of their new-born child, Harvey and Pattison [9] found that fathers recalled the event as clear and negative, characterised by fears over their partner and child, and lack of support postnatally, which led to them displaying PTSD-like symptoms. These studies emphasise the importance of considering the impact on men of what has often been viewed as primarily a matter for women.

In a cross-sectional study of fathers who witnessed ‘normal’ births on maternity wards in Belgium, Eggermont, Beeckman, Van Hecke, Delbaere and Verhaeghe [10] found that fathers’ need for information during the birth was greater than their need for involvement. Fathers required information around the process of birth, medical equipment used and their involvement in labour. This varied by educational level and parity such that fathers with a higher education level reported a lower need for information, as did fathers who had experienced birth previously. However different levels of need (or support) may be required for traumatic births.

There is limited research around the experiences of fathers regarding pregnancy and childbirth, likely due to their historical nonattendance in the delivery room. King [11] noted that prior to the 1970s, birth tended to be a female-only concern. Early theories of attachment downplayed the role of the paternal caregiver in childrearing [12, 13]. This is reflected in UK Government legislation, whereby mothers are automatically issued with full parental responsibility, whereas fathers are given parental responsibility only when they are married to the mother, listed on the birth certificate or receive a parental responsibility order from the mother or court [14]. However, the presence of fathers at the birth has increased within the last few decades, with nearly 95% of fathers in England and 98% of fathers in Scandinavian countries now reported as attending childbirth [15]. Further, becoming a father is one of the most life-changing events a man can experience (Jomeen, [16]) suggesting it is imperative to understand the father’s perinatal experiences and their possible consequences.

However, despite fathers attendance at births becoming more common, evidence suggests that men still do not feel welcomed by perinatal health professionals, In an analysis of 62 studies of perceptions about Swedish healthcare professionals from 2000 to 2015, Wells [17] found that fathers felt excluded at prenatal clinics and consequently unprepared for the experience of birth and fatherhood. Across the perinatal stages, fathers feel that midwives are often disrespectful towards them [17, 18]. Fathers who reported being treated respectfully by healthcare professionals were four times more likely to describe the birth as a positive experience, as they felt that they had greater control, lower anxiety and believed their partner was safe [17]. Such findings in Sweden, considered a gender-equal society, suggests that experience of feeling excluded is likely to be experienced by fathers worldwide.

Wells [17] emphasises that fathers felt societal pressures to be an equal parent, but being treated as secondary made them feel unable to demand to the support and information they felt they needed. This had an impact on the division of parental duties and reflected the message from healthcare professionals that mothers are more important in caring for their children. Wells [17] also suggested that men may need more support than women in the transition to parenthood, as they are less likely to access support networks, and may develop unhelpful coping strategies to deal with the transition to parenthood [19]. These issues of gender inequality need to be addressed to help empower fathers for the benefit of all the family. Overall, evidence suggests that fathers do not receive much attention, information or support during the perinatal period.

The present study aimed to explore the following questions: what are the experiences of fathers who witnessed their partner’s traumatic birth; how do these experiences impact those fathers’ wellbeing; and what support did they receive during and following these experiences?

## Method

### Participants

A purposive sample of 61 participants was recruited. Inclusion criteria included being a UK-based father aged 18 or over and having witnessed their partner’s traumatic birth (single or multiple birth). The study was open to all fathers who perceived the experience as traumatic, rather than setting specific criteria regarding the intensity of that trauma. Exclusion criteria included: if the birth trauma experience was over ten years previously (as the issues mentioned may no longer be relevant under the current National Health Service, and because fathers whose partners had given birth more than 10 years previously were considered less likely to be able to recall the event in detail); or if the participant’s partner and/or child had died as a result of the experience (as this was considered unethical in an online study as the participants might need additional specialist support that could not be offered).

Potential participants were invited via social media advertisements (Twitter, Facebook and LinkedIn) targeting mental health support groups and national perinatal mental health charities. Those adverts included a link to online portal which described the study and invited participation. This recruitment method meant participants were able to access the necessary support they may have required during (and following) their completion of the study from those organisations. As a result, a wider audience could be reached. Participation was voluntary and anonymous.

**Fig. 1:**
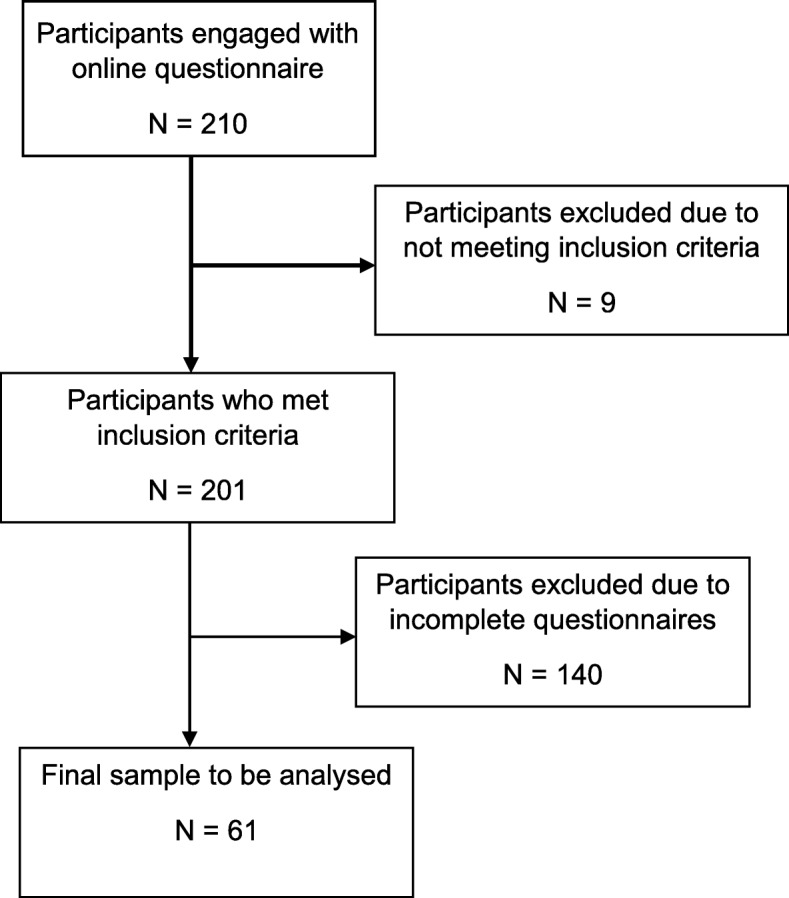
Flowchart of participant engagement

Nine participants were excluded because their experiences took place more than 10 years ago or because they were from outside of the UK. Flow of participants during the study is described in Fig. 1. Despite the specific inclusion criteria, the variety within the sample allowed for the reporting of a wide range of experiences. This contributed to the understanding of the similarities and differences of each individual participant’s trauma that may have been lost if the study had focused on a specific traumatic birth, for example, new-born resuscitation [9].

### Study design

The study was a qualitative questionnaire study. This is a well-established method for gathering information about sensitive topics, as it maintains participant anonymity [20].

### Procedure

An online (anonymous) qualitative questionnaire was created using Qualtrics™ to explore participants’ experiences and feelings of their partner’s traumatic birth. Given the nature of this subject, the questions posed were assessed for relevance and sensitivity by experts at Make Birth Better, a highly respected UK birth trauma network comprising perinatal health (and mental health) professionals. These experts were also a source for signposting participants for support if required. The questionnaire consisted of 20 open-ended questions and took up to an hour to complete. It focused on pregnancy (how the father felt when he became aware of his partner’s pregnancy, his involvement during the pregnancy), the birth experience (how he felt when his partner went into labour, what happened during the birth to him and his partner, whether he had received antenatal preparation for the birth, whether he understood what was happening, how in control he felt) and postnatal experiences (how he felt after the birth, what changes he expected after the birth, the extent to which what he witnessed at the birth had come back to his mind, whether the birth had affected his day-to-day life, whether he had had the opportunity to talk to someone about the birth, how the birth trauma had affected his mood, how it had affected his relationship with his partner), as well as the levels of support the participants received at each stage. The questionnaire is provided as supplementary online material.

Access to an online portal for the study was distributed on social media through an anonymous link (as described earlier). The entire process of providing information about the study, giving consent, participation and debriefing was undertaken through this online portal. Initially, potential participants were presented with information, which explained the purpose of the study, what participation would entail and the inclusion criteria. Contact details of the study team were provided in case participants required further information. Details were provided regarding where to seek support if this was required. The main source of support was *Make Birth Better* (https://www.makebirthbetter.org/). Respondents were then invited to indicate whether they consented to participate. While consent is usually assumed when participants answer questions in anonymous surveys, this was specifically sought to ensure that participants fully understood the sensitivity of the study. Once consent was given, participants were presented with the study questions. Participants were also reminded that they could discontinue if they found the questionnaire distressing. After completing the questionnaire, participants were presented with debriefing information, through the online portal, further highlighting sources of support and providing more detail about the study. Traumatic birth was defined as “*physical and emotional suffering during birth that resulted from either complications, physical injury or negative reactions during the birthing experience*”.

### Data analysis

Thematic analysis was used in order to allow for the complexity of the data as individual and collective accounts. The six stages of thematic analysis were followed [21]. The first author familiarised herself with the data collected by reading and rereading the transcripts. She then generated initial codes and combined them into broad themes. These were then refined and reviewed with the help of the second author, defined and validated and reported in a theme table. The first author was a young, childless female. This might have made it harder for her to understand the perspective of the participants (men experiencing childbirth). However, discussion with the third author, a man who is an expert in perinatal mental health and had experienced his partner’s normal childbirth, confirmed that the themes were relatable from a father’s perspective.

A reflexive diary was completed through all stages of analysis to identify possible misconstructions of themes and create transparency in analysis [22].

### Ethical considerations

The study was approved by Bournemouth University (BU), Faculty of Science and Technology, Ethics Committee (BU Ethics ID 12939). The study focused on sensitive topics that included birth trauma and mental health, which many of the participants may not have spoken about before. Therefore, participants’ right to withdraw from the study was highlighted. Participants were told to write only what they were comfortable with sharing to reduce potential distress. Participants were also encouraged to access support they already receive or to seek further support for their experiences. The responses were kept anonymous to protect confidentiality and to give participants the chance to speak freely about their experiences.

## Results

Participants came from all regions of the UK. Participant characteristics are reported in Table 1. One participant reported having twins, and three reported currently expecting another child. It was the first pregnancy for 81.9% of participants, and all participants were married.

**Table 1:** Participant data

Participant data	Mean ± SD	Range
Current age	36.6 ± 6.3	24–51
Age at time of trauma	33.8 ± 5.3	23–36
Time since birth trauma (years)	2.8 ± 2.0	
No. of children in family	1.6 ± 0.7	1–4

**Fig. 2:**
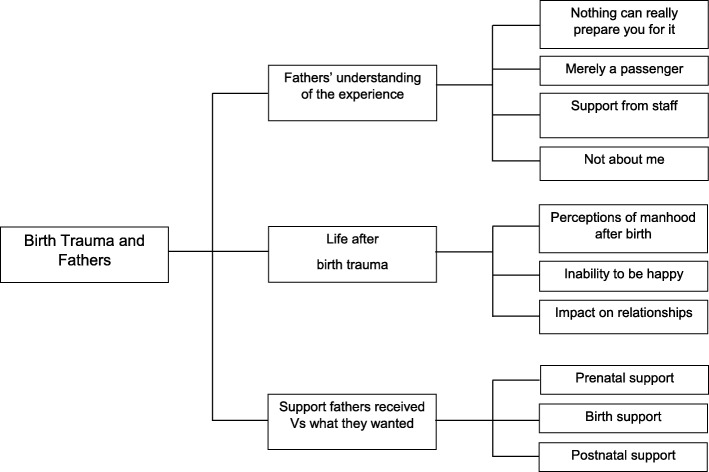
Flow chart of themes explored

The data analysed emphasised the situations that contributed to or protected from the birth trauma experience. Three main themes were identified: ‘fathers’ understanding of the experience’; ‘life after birth trauma’; and ‘the support fathers received vs what they wanted’. The flow of themes is demonstrated in Fig. 2.

### Fathers’ understanding of the experience

This first theme identifies the participants’ experiences during the birth of their child. This includes what participants believed contributed to their trauma experience as well as potential protective factors. This theme had several sub-themes: nothing can really prepare you for it; merely a passenger; not about me; and support from staff.

#### Nothing can really prepare you for it

Participants felt that they were not adequately prepared for the experience. They perceived preparation as information gained from antenatal classes and from healthcare professionals during the experience. Information was either not given or not provided to a standard of satisfaction. For example, one participant said “At no point was there any explanation to either my partner or myself to calm the situation.” (P16).

Information given at antenatal classes also failed to prepare fathers for traumatic birth. Participants felt they focused more on standard deliveries and lacked detailed coverage of potential negative situations. For example, “They briefed us on the basic pain relief options for labour and the basics of a birth plan. These seem almost laughable in hindsight.” (P2), and “The antenatal classes are too positive and preparation for all eventualities was poor.” (P20).

Participants explained that they were unable to understand what was going on around them due to lack of information or time to explain during the emergency. As a result, some reflected that the experience of traumatic birth may not be something you can prepare for: “To be honest, I don’t think anything could have prepared me for what happened.” (P3).

The participants knew when something was wrong during the birth experience, but they were not involved in decision making or in relaying information. Often, they were also left alone which contributed to the trauma, as one participant described “When our son was taken away there was no one to ask what had happened to him for quite a while.” (P1).

Overall, participants believed that they had not been properly made aware or given the opportunity to make themselves aware and prepared for the likelihood of a complicated birth, from antenatal classes and from staff during the experience. However, they recognised that it is not always possible to be prepared.

#### Merely a passenger

A lack of control during the complicated birth experience was described as a major contributor to the trauma. Participants either felt they never had control, or control was taken away from them by the situation and/or staff. For example, one participant explained “I wasn’t involved. Merely a passenger.” (P60), and another said the lack of control made him feel as if: “I had lost all control of the situation, which scared me to death.” (P10).

If any control was reported during the experience, it was from trying to help their partner, as one participant mentioned: “I did not feel in control at all, but I did feel very involved in looking after my partner’s welfare.” (P40).

Fathers who reflected more positively believed they had some level of control (even if small), which could be considered a protective factor against later developing mental health problems. While they spoke about having little control and finding it traumatic, they also noted how they probably did not want any control in that situation: “I knew I had no control and I did not feel involved. I didn’t think it appropriate that I was involved to be honest.” (P4); “Felt I had no control at all as there was nothing I could do to fix the situation just had to wait for the doctors to sort things out.” (P52).

In conclusion, this theme details how control featured in fathers’ experiences, how they attempted to create control by looking after the interests of their partner, while also exploring how having little control in this situation appeared to be most appropriate for fathers.

#### Not about me

This theme explores the roles fathers believed they had during the birth experience and their treatment as a result of being male. During the pregnancy and birth experience, participants suggested that being male may have impacted their treatment by professionals, family and friends, based on societal expectations around birth and the role men play in general. Participants mentioned not being listened to and their questions not being answered.

Participant 7 described how he felt regarding his role during the birth: “I felt mostly like a spare wheel to be kept out of the way.” (P7) Participants often felt that their presence in the experience was not acknowledged: “No. I’m the male. My presence was often not acknowledged let alone my feelings.” “The midwives weren’t very helpful after the birth as my wife developed depression. They ignored my concerns.” (P9) and; “However, not being made to feel like the enemy, a useless caveman that has thoughtlessly impregnated this innocent girl, would be a start.” (P13).

When fathers felt acknowledged, it was only as they were there to support their partners: “I remember feeling very emotional and almost breaking down when they wheeled her off, but quickly pulled myself together as I knew she still needed me to be strong and upbeat.” (P51).

This treatment controlled the fathers’ views of themselves and sometimes prevented them from accessing and seeking help when needed, in some cases leading to further problems long after the birth. Participant 47 explained: “I never would of brought it up to anyone even my wife how could I possibly tell her how traumatised I was when she’s the person that had the ordeal of having a baby.” (P47).

Overall, participants’ experiences of being male in this female dominated experience meant that they felt unimportant (like a spare wheel), with their role in supporting their partner. As a result, they felt they could not explore or discuss their thoughts and feelings during and after the experience.

#### Support from staff

When participants reported that staff were calm and communicated with the couple, this appeared to ease the father and act as a protective factor in their overall view of the experience and how they felt after the birth, as described by participant 26: “as the midwives and consultants were extremely calm, clear and communicated every stage with us.”*.* This was also reflected by participant 51: “While, what seemed like muted panic or urgency was erupting around my wife, he [surgeon] calmly explained what was going on and what would happen. Not much of what he said went in, but his calmness was infectious.”. This behaviour from staff helped fathers to feel calmer about the situation and therefore more relaxed.

Unfortunately, this level of support was not always possible, in some cases due to system issues:

“Unfortunately, the shift changes right before things escalated, so we didn’t have much opportunity to build a rapport with the new midwife.” (P2).

### Life after the birth trauma

This theme explores the participants’ postnatal experiences, including expectations of and from fathers, their mental health and the way in which their birth experiences impacted their relationships. Sub-themes include perceptions of manhood after birth; inability to be happy; and impact on relationships.

#### Perceptions of manhood after birth

This sub-theme explores changes in the participants’ roles going into fatherhood and how this impacted their health. Some fathers found that as a result of the traumatic birth, they became the primary caregiver for their child and sometimes their partner, as this quote shows: “We didn’t expect the difficulties we would face with my wife’s recovery. She ended up with a few issues and I spent a bit of time off work looking after her.” (P12) Participant 13 also reflected on his experience of becoming the primary caregiver for his child and partner that eventually impacted his work role and friendships: “The trauma of my son’s birth put me immediately into what felt like sole-responsibility for my entire family 24/7, ultimately excluded me from colleagues and friends and I struggled to cope.” (P13).

The trauma also impacted the participants’ expectations of fatherhood, where they felt that they did not meet the standards or expectations that society had given them. For example, they did not experience an instant bond with their child and in turn felt guilt and shame about themselves as fathers:

“I expected to feel an instant bond with the baby and I didn’t. I came to have a very strong bond with my daughter and I think not getting it initially was a combination of an unrealistic expectation of a romanticised version of what it is to have a child and the situation I was in. Had my wife been fine I wouldn’t have felt so conflicted and it may have come easier at the start.” (P57).

Participants also explored their role of being a ‘man’ and what their experiences meant in a female dominated experience. This also impacted how they coped with their experiences postnatally. They saw being a ‘man’ as being quiet and avoiding discussion of their experiences: “A man’s role in the birth is nothing to talk about really. Be quiet and man up, etc,” (P11). This was partly because they were aware of their partner’s trauma and believed it was greater than their own: “I chose not to think about it for some time; as a man, it does feel churlish to go on about your trauma when the female involved has this harrowing experience happening within her own body.” (P13).

However, participants also acknowledged the stigma around discussing emotions with other men, and not wanting to burden their friends, preventing them from speaking up and seeking support. For example: “As a lad it’s not easy to talk to male mates. I’m sure they’d listen but I don’t want to be a burden or a downer.” (P31).

In conclusion, this theme identifies how preconceived expectations of birth and the postnatal experience for men as men and fathers can contribute to feelings of unimportance around their own experiences. This in turn, leads to less support seeking and reinforces these expectations.

#### Inability to be happy

Participants described how the postnatal period affected their daily lives and how the trauma still affected them in the present day. Traumatic births negatively affected their mental health during the postnatal period, leading to difficulties coping with everyday functioning: “Upset, distressed and unable to cope very well. It was a feeling about the fragility of life during the birth and the overwhelming nature of the birth and the subsequent days.” (P14) Some participants experienced the development of mental illness in some cases: “… develop [ed] OCD within weeks of the birth. That might have been about the life changes that becoming a father involved but it might be rooted in that birth experience too.” (P1).

Most participants reported feelings and experiences during and after the birth trauma that can be linked to symptoms of PTSD. Participant 47 was diagnosed with PTSD and described flashbacks, triggers and avoidance: “I had flashbacks that seemed so real it was like I was there again, I deal with it better now but going back to the maternity wing of that hospital caused great anxiety, walking past the theatre doors etc the sound of a heart rate monitor sets me off even when it’s on the TV, I can’t watch things like Call the Midwife or One Born Every Minute or anything involving childbirth on the TV either.” (P47). Participant 60 also discussed flashbacks that were directly linked to the birth experience: “I regularly have flashbacks and see the number 61 on the heart rate monitor in my mind*.”* (P60).

Overall, participants described a range of mental health problems in the days and weeks following the birth, with some incidences leading to mental health disorders, which continued to impact their lives up to the present day.

#### Impact on relationships

This theme explores the impact the traumatic birth had on the fathers’ important relationships with their partner, children and friends. Most participants reported that the experiences brought them closer to their partner. Going through such a traumatic experience, including the fear that they could have lost their partners, created a stronger bond between couples: “Maybe there’s an extra layer to us as we survived such a difficult experience and we know that whatever happens in life we’re unlikely to encounter anything as testing.” (P3); “It’s made us closer because I know I could have lost her.” (P8).

However, many fathers discussed struggling to form a bond with their child (and feeling guilty): “I wanted to be with my wife but I felt guilty that I didn’t want to be with the baby. When I was with the baby I didn’t feel the level of love that everyone says you do and I felt guilty about that. I was just really confused and frightened.” (P57) Participants also expressed having fears around more children. “Only in the way that I’m reluctant to have sex in case she accidentally gets pregnant.” (P51) These feelings also manifested as difficulty celebrating their children’s birthdays, and fears about emotional intimacy with their partners, which impacted their relationships long term: “We don’t talk about what went on that day, each birthday is very difficult and not a celebration.” (P16).

In some cases, participants also felt this negatively impacted their relationships with friends, due to others lacking comprehension of the situation. As one participant explains: “I tried to talk to my friends, but most of them have no idea of children, or what it involves, so it’s hard for them to relate.” (P10).

Overall, negative birth experiences made the relationship with partners stronger in some cases. However, it also had a negative impact on partner relationships through affecting bonds with their children and leading to avoidance of physical and emotional intimacy. Relationships with those outside of the family were also negatively impacted in some cases, as friends did not share the same experiences and could not understand.

### Support fathers received vs what they wanted

This theme consists of the levels of support fathers felt they received either personally, as a couple or for their partner, in relation to what support they believed would have been beneficial. It is split into three sub-themes that explore different time periods of the experience: prenatal support; birth support; postnatal support.

#### Prenatal support

During the pregnancy, participants reported receiving a good level of support, where all concerns raised were answered: “As much support as was needed. The pregnancy was pretty good to be honest so we didn’t require any additional support but whenever we had questions then we got the answers.” (P11) However, they did note that antenatal classes were very much tailored towards mothers. For example, when participant 51 was asked about the support he received prenatally, he said “Personally, none. But my wife was well looked after and supported. However, I never felt left out or unwelcome.” (P51).

However, in retrospect, participants felt antenatal classes only discussed normal births, which they felt created an idealised experience. Participants reported wanting antenatal classes to expand on the breadth and depth of the childbirth experience, to cover departures from the norm: “… in retrospect, I see these as “idealised“ birth training classes. Breathing exercises, birth pool options, stages of normal labour etc,” (P29) and “More information on what a birth plan could involve, and learning about how variable each pregnancy is, as any change from the norm made us quite nervous.” (P10).

Furthermore, participants felt that ideally antenatal classes would include more information around being included as a father. For example, one participant felt that: “I should have been more included from the beginning. A mother and father should both complete questionnaires regarding depression and a father should be asked how he is feeling or if there is anything that he wants to ask or doesn’t understand. These questions are solely aimed at the expectant mother whilst a father has to butt in and speak during a conversation he is only there to witness.” (P21) Participants also explained the importance of peer support for partners during the prenatal period: “Peer to peer support from other fathers, an antenatal session for partners on supporting birth & the early days and information tailored to males (language, imagery & context).” (P55).

Overall, fathers felt they could have been provided with more information and involved more in sessions such as antenatal classes during the prenatal period.

#### Birth support

Participants described the levels of support varying throughout the birth as generally less positive than prenatal support. For example: *“In the 70ish hours before the actual birth support levels went up and down.”* (P1).

However, the fathers often reported that this was understandable due to the emergency situation. They were aware that the lack of support for them meant that their partners were being saved: “Emotionally/mentally, I remember receiving very little support. Can’t fault them too much though - they were busy saving my partner and baby!” (P30).

Nevertheless, participants would have liked to be informed and involved throughout the birth. They felt that being involved would have made them feel more useful: “I would have liked to have had someone to stay with me or at least pop in regularly to see if I was okay and to tell me what was happening with my wife.” (P8) and “To be made to feel like a useful part, to be involved and kept updated on what was going on and why” (P58).

In some cases, they felt that the lack of support received contributed to them labelling the experience as traumatic: “More support in making decisions regarding this [implications of delivery] – this was a major contributor to the trauma of our delivery.” (P2).

This theme explored how a lack of support was perceived by participants as a contributor to their traumatic experience. Participants wanted to be involved in making decisions and wanted someone to support them but were aware of the need to focus on the mother.

#### Postnatal support

Participants discussed support received in the months after the birth, plus what they felt would have been beneficial. Many fathers felt there was minimal support for themselves or their partners: “We had some follow up care for our daughter and 2 discussions with doctors regarding the delivery and what went wrong. These were minimally informative.” (P2). Some participants sought professional help as a result: “I paid for private therapy. My wife had no follow up from her mental health team except a 20 min meeting alone with them …” (P4).

Participants felt a chance to discuss their experiences and receive explanations after the event would have helped them make sense of the situation. As participant 2 explains: “More information from doctors about what happened to us – and an interview, possibly with administration or some other body to help us understand what happened and to give feedback – this we would not get unless we took the initiative to pursue such an opportunity. I think this kind of debriefing should be standard when things go outside the parameters of a normal delivery.” (P2) Many participants reported wanting support from other dads, which could be provided via support groups, even mental health support/therapy themselves: “Maybe I could have joined a group of dads to discuss.” (P42); “Advice from other dads.” (P56).

However, participants acknowledged that men do not always seek help: When asked how much support he received, one father replied “Very little, but I could have sought more help, so I put no blame there.” (P30) Participants suggested that help could be explicitly directed and made easier to find in an attempt to break down barriers that prevent men from seeking help: “Perhaps it would of been nice to be told about the support rather than having to seek it.” (P43).

This final theme described the lack of or minimal amount of support for fathers in the postnatal period. Fathers felt that support could be given in the form of debriefs, ‘dad groups’ and support being made explicit to encourage fathers to seek support when they needed it.

## Discussion

The current study explored narratives of 61 fathers who had witnessed their partner’s traumatic birth. The themes that emerged reflected fathers’ experiences of traumatic birth, how these experiences impacted their mental health, and fathers’ feelings regarding support they received.

### Findings and interpretation

Participants felt that nothing could really prepare them for a traumatic birth, echoing the findings of Harvey and Pattison [9], who explored the experiences of fathers who witnessed their child’s resuscitation. Although fathers had been aware that a traumatic birth was possible, and had tried to prepare themselves for all eventualities, they had not considered the possibility of resuscitation so did not access information on that. In the current study, many fathers reported attempting to prepare for the birth by attending antenatal classes, and appointments with doctors and other perinatal health professionals. However, fathers still felt unprepared as they believed the information they received focused on ‘normal’ births, leading lack of awareness of the possibility of a traumatic birth. Eggermont et al., [10] also found that a father’s need for information outweighed his need for involvement during the experience, and he required less information if he had experienced a birth before. Many fathers in the current study reported similar experiences of their first childbirth. However, Eggermont et al., [10] focussed on ‘normal births’ meaning only limited comparisons can be made. Further, in a study into women’s traumatic birth experiences, Modarres et al., [3] found that a lack of information contributed to the trauma, which was supported in the current study.

The fathers in the current study reported feeling a loss of control, which they linked to being left out of decision-making and having limited access to information during the birth and saw this lack of control as a major contributor to their perceived trauma. Fathers who reflected more positively on their experiences noted that they had some control during this experience. A sense of control has been identified as being important for men in Western societies [19]. This research also corroborates findings that loss of control in the birth experience is a contributing factor for experiencing birth trauma in women [6, 9, 23, 24].

Fathers’ experiences with staff greatly influenced their perceived trauma. For example, more positive experiences with staff appeared to act as a protective factor against low mood postnatally. On the other hand, participants associated negative experiences with staff (such as not being listened to) as contributing to the traumatic experience. Fathers in the current study felt that being a man in the birth experience affected how healthcare professionals treated them, reinforcing that they should not be involved as this was a woman’s domain, in line with previous evidence regarding fathers’ negative experiences with perinatal healthcare staff, such as being treated as less important than mothers (e.g. Poh, et al. [9, 17, 18]). This underpins the concept that fathers feel that they are ‘secondary parents’, placing further stereotypical roles and responsibilities on the mother. This may also explain the lack of research into fathers and birth trauma.

However, the reasons for fathers not being given the support they need during childbirth may relate to attitudes within Western societies, where gender roles demand that men should be strong and in control, not needing help with their problems [19]. Women tend to speak more freely about how they feel regarding mental health, whereas men’s behaviour may be more of an indication that they are experiencing poor mental health, rather than what they say [25]. For example, higher rates of substance use (nicotine, alcohol and drugs) have been documented in fathers than mothers during the post-natal period. Wilhelm [25] suggests this could be due to men having poorer emotional health literacy than women, meaning they are less likely to report their feelings and seek help. Also, men tend to regard frequently utilising healthcare resources as a female trait [26]. Regardless of the reason, fathers’ poor mental health tends be overlooked by healthcare professionals.

Our study found that perceptions of masculinity limited fathers from talking about their experiences and may have prevented them from seeking help. Vogel et al. [19] reported similar findings. Previous studies have shown how fathers’ coping strategies were influenced by their beliefs about masculinity [8] and how men experience psychological distress when they fail to live up to internalised beliefs of masculinity [27]. Fathers may struggle with overcoming trauma and seeking help. Participants in our study reported struggling with perceptions of responsibility in their new role as a father, which may have caused them to keep quiet about their struggles and evade the topic of birth, even avoiding intimacy with their partner to prevent another birth. We suggest that these male coping styles fall in line with gender roles that are primarily emotionally restricted. These negative coping styles can contribute to the development and maintenance of PTSD [28].

The impact of traumatic birth had serious implications for some of the participants in the current study, with mental health issues such as depression, anxiety, postnatal stress, obsessive-compulsive disorder (OCD) and PTSD symptoms being described. Similar mental health difficulties and relationship problems for fathers following their traumatic experiences have been reported elsewhere [6–9]. Fathers also talked about how the relationship with their infant had been affected. Previous research by Ramchandani, et al. [29] showed that children’s long-term mental health and behaviour can be negatively affected when their father had a mental health problem in the perinatal period.

Fathers in the current study reported that they were often neither offered nor sought support after witnessing their partner’s birth trauma, and were not aware of what support was available. This may have been due to men taking longer than expected to realise they had a problem, and consequently experiencing delays in detection of health issues (as was found by Wilhelm [25]). Many fathers reported receiving a short debrief by practitioners following the trauma, but were left feeling they had not been given the time and space to process the event effectively. Fathers often expressed concerns over their partner’s mental health which perinatal services failed to address effectively. This in turn affected the fathers’ own wellbeing. Overall, participants wanted to be offered an opportunity to talk to someone (either professionally or to a peer) or to have access to therapy or counselling for themselves and for their family. However, it is important to note that debriefs are not necessarily effective in themselves at addressing the effects of a traumatic birth. Sheen and Slade [30] found that postnatal debriefs were ineffective in reducing post-traumatic stress or depressive symptoms and should not replace targeted interventions. Despite this, the participants in the current study suggested that receiving some information about what had happened would assist with their own understanding of the process.

### Implications for clinical practice and policy

Healthcare professionals need to be aware of factors that may prevent fathers from seeking help and professionals from providing support, and be ready to direct the fathers to where they can receive help and support. The fathers in the study stated that they felt ill-prepared for the possibility of birth trauma, and poorly informed about potential impact (for their partner and themselves) following that experience. This implies that adequate information during the experience and aftercare from birth trauma is key. Possible improvements to clinical practice could include offering parents the opportunity to understand what happened in the birthing process and be directed to opportunities for support if needed. This could be provided by the voluntary and charity sector, focusing on interventions such as peer specialist help, online support groups, or targeted interventions such as postnatal PTSD therapy.

Fathers in this study indicated that they felt excluded by postnatal health professionals (such as midwives and health visitors), claiming that those services were focused mostly on the mothers. This suggests it is essential that healthcare professionals recognise fathers as primary parents, in line with mothers, so that they can receive the necessary support.

Many fathers discussed how the traumatic birth impacted the bond with their child. Services need to be provided to help fathers develop that relationship. If further or more complicated issues are identified during the debrief, parents could be referred to specialist NHS interventions. Follow-up screening and referral needs to be ongoing, as trauma symptoms can sometimes be delayed for several months [31].

### Strengths and limitations

The current study generated rich and broad data based on the narratives of 61 fathers reflecting on their experience of witnessing their partner’s traumatic birth. By preserving participant anonymity through online questionnaires, this study elicited detail that might otherwise have been withheld. This is especially important as the data showed how difficult men find it to discuss birth trauma. However, questionnaires may limit the extent to which additional information can be generated. It could be useful for future studies to consider interview techniques that maintain anonymity, such as online interviews in real time. Such interviews might permit further probing of important perceptions.

By advertising this study online, it was possible to raise awareness about birth trauma and men’s mental health. However, the fact that participants were recruited via social media linked to mental health support and national perinatal mental health charities, suggests the participants had engaged with mental health support at least to some degree, and fathers who had not engaged with mental health support services were likely underrepresented. This issue might be overcome by engaging men who are part of other fatherhood groups, perhaps not necessarily related to mental health support.

Also, all participants reported that they were married, suggesting a lack of engagement of unmarried fathers. Future studies could explore whether there any differences for outcomes based on marital status. Further, 140 participants started the questionnaire but did not finish answering the questions. It is possible that the length of the questionnaire may have been a barrier to participation. Equally, some participants may have found the questions too distressing to answer. Future studies could pilot a range of questions to ensure that they gather the information needed without causing undue distress.

A further limitation is that the questionnaire was subject to recall bias. Participants, particularly if the traumatic birth occurred a long time ago, may not remember it accurately, or their memories may have become distorted over time. The current study excluded participants if the birth trauma occurred more than 10 years previously. Future studies may consider whether that period is still too long.

### Implications for future research

Future studies could explore how fathers’ perceptions differ according to the severity of the birth trauma encountered by their partner. In this study, we were careful to define birth trauma’ as any experience that might be considered to be traumatic. However, situations where there has been a more obvious threat to the welfare of mother and child may present different outcomes to other stressful birth events. Such an examination could potentially highlight how different types of traumatic experience might trigger poor mental health outcomes. Studies could also directly compare normal and traumatic births in a single study. Future research could also formally assess the impact of traumatic birth on fathers’ mental health using appropriate psychometric instruments.

Other studies may also benefit from comparing the birth trauma experiences of men and women together, in order to identify similarities and differences in traumatic experiences. Following on from Iles et al. [7], who explored similarities between mothers’ and fathers’ post-traumatic stress post-birth, it would be beneficial to explore potential differences between mothers and fathers as active participants and active/passive bystanders to the experience. Further research is also needed to explore fathers’ concepts of masculinity in the context of birthing, as current evidence may be based on outdated perceptions.

Research into effective interventions or preventative measures for fathers and their families may be crucial to fully address this area of research. This includes developing a deeper understanding of the barriers that prevent men from seeking help, as well as why practitioners may not offer support to fathers. By encompassing all perspectives from a traumatic birth, a better understanding can be achieved, resulting in preventative action, or, where this is not possible, effective aftercare.

## Conclusion

Our findings suggest that traumatic births can have a profound impact on some fathers, which can ultimately negatively impact their relationship with their family and their own mental health. Many fathers in this study reported feeling unable to access support during and following a traumatic birth, which were related to perceptions of the birth and treatment by perinatal staff. Many fathers in this study spoke about the impact that the traumatic birth had on how well they bonded with their new child. Several recommendations for clinical practice merit consideration: more education is needed for perinatal health professionals about the role of the father in the perinatal period; strategies are needed to encourage fathers to seek help; screening needs to be put in place to identify fathers who are most at risk of poor mental health; and services need to be developed to ensure fathers receive appropriate support for their own mental health and help nurture the father-infant relationship.

## Supplementary information

**Additional file 1.** Questions for Birth Trauma Questionnaire.

## Data Availability

The datasets used and/or analysed during the current study are available from the corresponding author on reasonable request.

## Abbreviations

PTSD: Post-traumatic Stress Disorder
OCD: Obsessive-compulsive Disorder
