# Supplementary material for: Be quiet and man up: a qualitative questionnaire study into fathers who witnessed their Partner’s birth trauma
Source: BMC Pregnancy Childbirth. 2020 Apr 22;20:236. doi: 10.1186/s12884-020-02902-2 (PMC7179006; doi:10.1186/s12884-020-02902-2)
Supplement: Supplementary file 1 — Additional file 1. Questions for Birth Trauma Questionnaire. [file 12884_2020_2902_MOESM1_ESM.docx]

Questions for Birth Trauma Questionnaire

At the top of every page, participants will be reminded to only divulge as much as they are comfortable with. They will also be reminded that if they feel distressed because of the nature of the study, they should stop and withdraw and there will be a link to the birth trauma association web page.

Before questionnaire:

Before the questionnaire starts, we would like to ask some basic information about yourself:

What is your age:

What was your age at the time of the birth trauma?

Where do you live: Choices will be regions of England

Relating to the birth trauma, was this your first-time pregnancy? Y/N

How many children do you have?

What is your relationship to the person who you shared the traumatic birth with?

Married/cohabiting/relationship, but not living together/other (please describe)

For the purpose of this study, the questions will refer to this person who you shared this experience with as your ‘partner’, but your answer here will be noted for analysis.

Section 1:

Birth Trauma in this particular study is defined as distress caused during birth possibly due to a complication, physical damage or negative emotions arising during and for some time postnatal.

1. Have you witnessed birth trauma with your partner?

YES or NO

If NO – questionnaire stops completely for that participant. Shown debriefing sheet.

2. Have you experienced the loss of a partner and/or child because of a traumatic birth?

If YES – the questionnaire stops completely for the participant. Shown Debriefing sheet.

If NO – Next question

Section 2:

1. Starting from the beginning, can you describe how you felt when you became aware of your partner’s pregnancy?

2. How involved were you during her pregnancy (e.g. did you attend antenatal classes, scans, midwife appointments, etc.). Please explain.

3. What support (if any) did you receive from healthcare professionals for this pregnancy?

4. What support would you have liked to have received?

Section 3:

1.. How did you feel when your partner went into labour?

(Where were you when it happened, how did you hear about it, what did you do?)

2. What happened during the birth, to your partner and to you?

2a. Did you receive any antenatal preparation for your partner’s birth and how did this preparation help you during your partner’s labour?

2b. Did you understand what was happening and can you explain why?

2c. How in control/involved did you feel and why was this?

2d. What support did you receive (if any) during the birth from healthcare professionals?

2e. What support would you have liked to have received?

Section 4:

1. How did you feel after the birth?
2. What changes did you expect/not expect to happen after this birth?
3. To what extent has what you witnessed at the birth come back to your mind? Please describe.

4a. Do you think this has affected your day to day life? If so, how?

4b. Do you feel you have had an opportunity to talk to someone about it? If yes or no, please explain why.

4c. Has the birth trauma affected your mood? If so, how

1. How do you think the birth trauma has affected the relationship you have with your partner?
2. What support did you receive (if any) after the birth from healthcare professionals?
3. What support would have liked to have received?

Is there anything else that you would like to tell us about your experience of a traumatic birth that the questions above did not address?
